# Early cartilage lesion and 5-year incident joint surgery in knee osteoarthritis patients: a retrospective cohort study

**DOI:** 10.1186/s12891-024-07225-3

**Published:** 2024-05-21

**Authors:** Liu Xiao-feng, Zhang Jin-shan, Zheng Yong-qiang, Wang Ze-feng, Xu Yong-quan, Fang Yang-zhen, Lin Zhen-yu, Lin Liang, Zhang Hong-peng, Huang Xiao-peng

**Affiliations:** 1 Department of Orthopedics, Jinjiang Municipal Hospital, Fujian, China; 2 Clinical Research Center for Orthopaedic Trauma and Reconstruction of Fujian Province, Jinjiang Municipal Hospital, Fujian, China

**Keywords:** Knee osteoarthritis, Cartilage lesion, Joint surgery, Association, Predictive value

## Abstract

**Objective:**

to investigate the association between cartilage lesion-related features observed in knee osteoarthritis (OA) patients’ first MRI examination and incident knee surgery within 5 years. Additionally, to assess the predictive value of these features for the incident knee surgery.

**Methods:**

We identified patients diagnosed with knee OA and treated at our institution between January 2015 and January 2018, and retrieved their baseline clinical data and first MRI examination films from the information system. Next, we proceeded to determine joint space narrowing grade, cartilage lesion size grade, cartilage full-thickness loss grade and cartilage lesion sum score for the medial and lateral compartments, respectively. Generalized linear regression models examined the association of these features with 5-year incident knee surgery. Positive and negative predictive values (PPVs and NPVs) were determined referring to 5-year incident knee surgery.

**Results:**

Totally, 878 participants (knees) were found eligible to form the study population. Within the 5 years, surgery was performed on 61 knees. None of the cartilage-related features had been found significantly associated with incident surgery. The results were similar for medial and lateral compartments. The PPVs were low for all the features.

**Conclusions:**

Among symptomatic clinically diagnosed OA knees, cartilage lesions observed in the first MRI examinations were not found to be associated with the occurrence of joint surgery within a 5-year period. All these cartilage-related features appear to have no additional value in predicting 5-year incident joint surgery.

**Supplementary Information:**

The online version contains supplementary material available at 10.1186/s12891-024-07225-3.

## Introduction

Osteoarthritis (OA) is a common chronic joint disease that is often accompanied by symptoms such as joint pain, morning stiffness, and restricted movement [1]. The structural changes in the joints caused by OA primarily include cartilage lesion, osteophyte formation, synovitis, meniscal extrusion or tear, subchondral bone damage, and ligament rupture. Pathological changes in cartilage have always been a focus of research, and many researchers use the degree of cartilage lesion to measure the progression of osteoarthritis [2]. In addition, various treatment methods for cartilage repair have been proposed, with the hope of delaying the progression of the disease or curing OA by stopping further cartilage damage or repairing already damaged cartilage [3–5].

However, to date, no medication has been proven to effectively prevent OA from progressing to the late stage. Many patients still end up needing joint replacement surgery. It is well known that joint surgery requires a significant amount of medical resources and healthcare funds, and the surgery often does not restore patients to their pre-disease state, not to mention the potential postoperative complications that can greatly reduce the quality of life for patients [1]. Therefore, being able to identify early those patients who will eventually require surgical treatment and intervening early to avoid the tragedy of entering the operating room is crucial [6, 7]. Previous studies have shown that factors such as patients’ sex, socioeconomic status, and physicians’ preferences are associated with the occurrence of joint surgery [8, 9]. However, there is little literature reporting whether early cartilage lesion in clinical practice indicates that the disease will progress to the status in need of surgical interventions in the coming years. Cicuttini and Wluka found that cartilage lesion could be used to predict future knee joint surgery, both studies used the same population but were published more than a decade ago and involved very few patients who underwent surgical intervention (only 18 patients in each study) [10, 11]. Therefore, the conclusions of these studies need to be further validated by larger-scale research.

In this study, we collected imaging data from knee OA patients when they underwent their first MRI examination in clinical practice. We conducted an association analysis between the cartilage damage related features on radiographs and MRI (i.e., joint space narrowing and cartilage lesion) and the risk of joint surgery within 5 years after the examination. At the same time, we calculated and evaluated the predictive values of these features for surgery within 5 years. We hypothesized that early cartilage damages observed in patients’ first MRI examination would be associated with 5-year incident joint surgery.

## Patients and methods

### Study population

Between January 2015 and January 2018, we identified patients who were diagnosed with knee OA and treated at our regional tertiary hospital. The hospital information system was utilized for this purpose. Our research study followed the guidelines and regulations established by the ethics committee of Jinjiang Municipal Hospital. The ethics committee of Jinjiang Municipal Hospital granted approval for data collection and the conduct of the current retrospective cohort study (Approval No. JJSYYYXLL2015001). Informed consent was obtained from all patients once they have administered in our hospital that their medical data may be used for scientific analysis but will be kept anonymous.

The cohort was formed by selecting knees that met specific criteria. To be included, knees needed to belong to patients aged 40 years or older, have reported knee pain, be confirmed to have radiographic knee OA (Kellgren & Lawrence (KL) grade > = 2) through radiographs, and have available magnetic resonance imaging (MRI) films. The baseline for this study was determined by the year the patient received their first MRI examination after being diagnosed with knee OA. Knees were excluded if they had suffered a knee injury causing structural damage after the baseline period, had any other knee joint diseases or forms of arthritis, if the patient had not undergone radiographs or MRI exams, or if the patient had a planned knee surgery at baseline. In cases where a patient had OA in both knees, we selected the knee that was first diagnosed with OA. If both knees were diagnosed during the same clinical visit, one knee was randomly chosen for inclusion in the cohort. For this study, knees that did not receive a radiographic exam in the same year as the MRI exam were excluded.

### Baseline patient data

Following the inclusion of a patient in the study, their baseline clinical data and imaging films were obtained from the health information system. The baseline clinical data comprised demographic information such as age, sex, BMI (body mass index), education, smoking status, affected side of knee OA, and the presence of OA in other joints. Additionally, the pain level experienced by the patients in the past week was assessed using the numeric rating scale (NRS) score, ranging from 0 to 10 (where 0 indicates no pain). This assessment was conducted at the time of receiving MRI examinations.

Two researchers, who were blinded to the patients’ clinical information, independently reviewed weight-bearing knee radiographs taken at the baseline. We assigned KL grades to the tibiofemoral joint using the KL classification system [12]. Furthermore, we assigned joint space narrowing (JSN) grades (ranging from 0 to 3) to the lateral and medial compartments, respectively. A JSN grade of 0 indicated no JSN, while grades 1, 2, and 3 represented minimal, moderate, and severe JSN, respectively. In cases where discrepancies arose, the two researchers held a consensus meeting to resolve differences and reach agreements.

### MRI scan and cartilage lesion assessment

All knees underwent imaging using a 3.0T MRI machine in the sagittal plane at our hospital. Two researchers, who were unaware of the patients’ clinical information, independently reviewed the baseline T1 and T2 MRI films. They utilized the standardized MRI Osteoarthritis Knee Score (MOAKS) system to assign grades for cartilage lesion size and full-thickness loss [13]. The final grades were made upon agreement by the two (intraclass correlation coefficients (ICC) range from 0.821 to 0.965).

In detail, we evaluated the cartilage lesion size and full-thickness loss grades for each subregion, consisting of six subregions for the femur and two for the tibia, as defined by the MOAKS system. Cartilage lesion size was graded on a scale of 0 to 3 based on the percentage of subregional involvement. Grade 0 indicated no involvement, grade 1 indicated involvement of less than 10% of the subregional surface, grade 2 indicated involvement of 10–75% of the subregional surface, and grade 3 indicated involvement of more than 75% of the subregional surface. In cases where multiple cartilage lesions were present within a single subregion, their sizes were combined to determine a single percentage. We then determined the cartilage lesion size grade for the medial and lateral compartments by selecting the highest grade among the corresponding subregions.

Similarly, the cartilage full-thickness loss grade was assigned on a scale of 0 to 3 based on the percentage of full-thickness cartilage loss within the subregion. Grade 0 indicated no loss, grade 1 indicated less than 10% involvement, grade 2 indicated involvement of 10–75%, and grade 3 indicated involvement of more than 75%. Likewise, we determined the full-thickness cartilage loss grade for the medial and lateral compartments by selecting the highest grade among the corresponding subregions.

Based on this, we calculated the cartilage lesion sum score by summing the cartilage lesion size grade and the full-thickness cartilage loss grade for both the medial and lateral compartments. As a result, the cartilage lesion sum score ranges from 0 to 6.

### Outcome measure

Upon reaching the 5-year mark, we contacted all patients to ascertain whether they had undergone any knee surgeries specifically for knee OA in the knee that had been examined during the baseline MRI. The knee surgeries encompassed total or unicompartmental knee replacement, arthroscopic procedures, and high tibial osteotomy. If multiple surgeries were performed on one knee, we considered the first surgery. To validate this information, we cross-referenced the patients’ medical care records within our hospital information system.

### Statistical analysis

Descriptive statistics were used to summarize the characteristics of the subjects in our study, and t-tests or Chi-square tests were used for comparing baseline characteristics between knees with and with incident surgeries when appropriate. Logistic regression models were constructed to examine the association between baseline JSN grade, cartilage lesion size grade, full-thickness loss grade, cartilage lesion sum score, and the occurrence of surgery over the 5-year period. This analysis was conducted separately for medial and lateral compartments. Crude odds ratios (ORs) and adjusted ORs (adjusted for baseline age, sex, and BMI) were calculated, along with their corresponding 95% confidence intervals (CIs). For MRI cartilage lesion grades, we additionally adjusted the analysis for baseline KL grade. The reference group for comparison in all features was baseline grade 0. Few knees were graded with JSN grade 3 at baseline, so we combined the JSN grade 2 and 3 when performing the analysis. The selection of covariates was based on their potential as confounding factors.

As a sensitivity analysis, we excluded knees that underwent arthroscopic cartilage repair surgeries within the 5-year timeframe and repeated the aforementioned association analysis.

Furthermore, we calculated the positive and negative predictive values (PPV and NPV) with their respective 95% CIs for baseline JSN grades and cartilage lesion grades in predicting the incidence of surgery over the 5-year period. To facilitate analysis, we categorized JSN grades and cartilage lesion grades into a dichotomous variable of grade 0 versus grade 1 or more.

Missing baseline characteristic data in knees were imputed using data from the latest clinical visit, i.e., age, sex, and BMI. However, 65 knees with missing values in the baseline NRS score were excluded from the descriptive analysis. The primary outcome measure, surgery information, had no missing values as we directly contacted the patients and most of the data were available in the healthcare information system.

All statistical analyses were conducted using the SPSS software (IBM, Chicago, USA), and statistical significance was defined as a *p*-value less than 0.05.

## Results

**Fig. 1:**
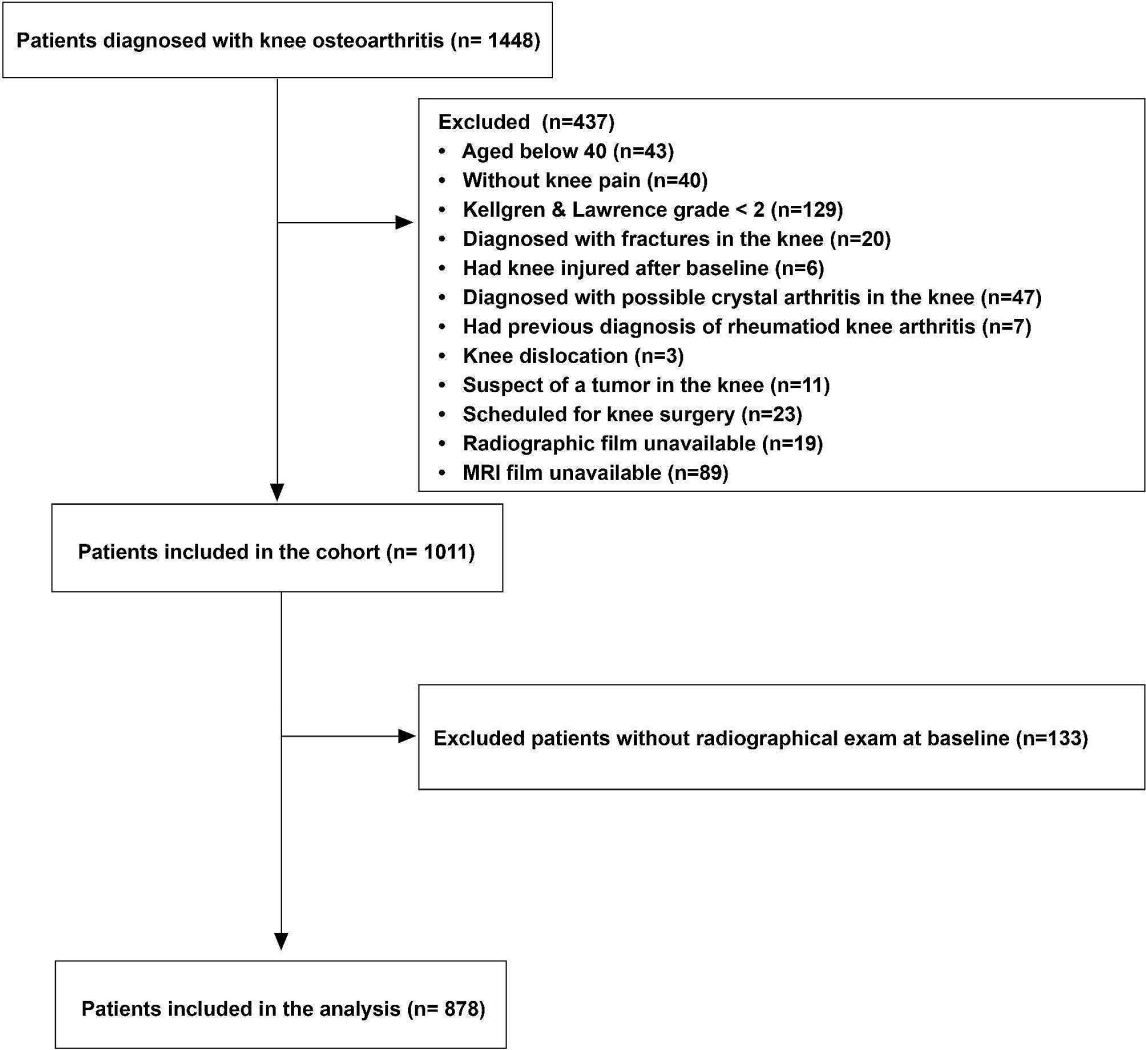
Patient inclusion procedure

In total, 1448 patients were diagnosed with knee OA and identified; of those 437 were excluded, the most common reason for exclusion was KL grade < 2 at baseline (*n* = 129). Thus, 1011 participants (knees) formed the cohort and further 133 knees were excluded for missing radiographic exams. See detailed patient inclusion procedure in Fig. 1. As a result, 878 participants (knees) were found eligible to form the study population with a mean (SD) age of 61 (8.7), 73% female and a mean (SD) BMI of 25.1 (3.6). Within the 5 years, surgery was performed on 61 knees; specifically, 23 knees had total knee replacement, 7 had uni-compartment replacement, 22 had arthroscopic procedures (for removing damaged cartilage, repairing degenerative ligament, meniscus tear, partial meniscectomy and debridement) and 9 had high tibial osteotomy. The baseline characteristics of the knees with/without surgery are presented in Table 1.

**Table 1:** Baseline demographic and radiographic characteristics of the knees received/did not receive surgery within the 5 years

Characteristics	Knees surgery (*n* = 61)	No knees surgery (*n* = 817)	*p* values
Age, years, mean (SD)	61.7 (8.1)	61.3 (8.6)	0.712
Sex, female (%)	48 (79)	596 (73)	0.328
BMI, kg/m^2^, mean (SD)	24.7 (2.6)	25.2 (3.6)	0.243
Bilateral OA, yes (%)	47 (77)	670 (82)	0.334
Education, high school or higher (%)	21 (34)	339 (41)	0.416
Smoking, n (%)	13 (21)	137 (17)	0.363
Have hand OA, n (%)	9 (15)	51 (6)	0.011
Have hip OA, n (%)	6 (10)	82 (10)	0.960
Time from OA diagnosis to enrollment, months, mean (SD)	53.1 (26.6)	51.6 (27.6)	0.665
NRS pain score, 0–10, mean (SD)※	1.7 (1.3)	1.8 (1.3)	0.823
Baseline tibiofemoral KL grade			0.098
2	49 (80)	562 (69)	
3	11 (18)	249 (30)	
4	1 (2)	6 (1)	

SD, standard deviation; BMI, body mass index; OA, osteoarthritis; NRS, numeric rating scale; KL, Kellgren & Lawrence

※65 knees had missing values in NRS score

### The association between baseline JSN and 5-year incident surgery

For lateral compartment, JSN grade 1 indicated higher risk of 5-year incident surgery, but the association was not statistically significant with a crude OR (95%CI) of 1.5 (0.6 to 3.5) and an adjusted OR (95%CI) of 1.5 (0.6 to 3.6). JSN grade 2 & 3 indicated similar and non-significant risk to JSN grade 0 with a crude OR (95%CI) of 1.0 (0.4 to 2.7) and an adjusted OR (95%CI) of 0.98 (0.36 to 2.7). For medial compartment, both JSN grade 1 and JSN grade 2 & 3 indicated higher risk of 5-year incident surgery, but the associations were not statistically significant (Table 2). Similar results were found in the sensitivity analysis after excluding 6 knees underwent arthroscopic cartilage repair surgeries (Appendix Table 1).

**Table 2:** Association of baseline Kellgren & Lawrence and joint space narrowing grade with 5-year incident knee surgery

	Surgery/without surgery, n/n	Crude OR (95% CI)	*P* values	Adjusted OR (95% CI)#	*P* values
**Lateral Joint space narrowing grade**					
0	6/103	Reference		Reference	
1	43/506	1.5 (0.6–3.5)	0.400	1.5 (0.6–3.6)	0.391
2 & 3	12/208	1.0 (0.4–2.7)	0.985	0.98 (0.36–2.7)	0.975
**Medial Joint space narrowing grade**					
0	2/70	Reference		Reference	
1	47/525	3.1 (0.7–13.2)	0.119	3.2 (0.7–13.4)	0.115
2 & 3	12/222	1.9 (0.4–8.7)	0.411	1.9 (0.4–8.6)	0.416

OR, odds ratio; CI, confidence interval

# Adjusted for baseline age, sex and BMI;

### The association between baseline cartilage lesion and 5-year incident surgery

For lateral compartment, neither higher lesion size or higher full-thickness loss grade indicated a higher risk of 5-year incident knee surgery (Table 3). Similarly, to medial compartment, some (e.g., medial lesion size grade 1 to 3) even implied a decreased risk of surgery although the associations were not statistically significant (Table 3). Sensitivity analysis results support these findings (Appendix Table 2).

**Table 3:** Association of baseline tibiofemoral cartilage lesion grade with 5-year incident knee surgery

	Surgery/no surgery, n/n	Crude OR (95% CI)	*P* values	Adjusted OR (95% CI)※	*P* values
**Lateral lesion size grade**					
0	11/138	Reference		Reference	
1	11/231	0.6 (0.2–1.4)	0.241	0.6 (0.3–1.5)	0.286
2	21/241	1.1 (0.5–2.3)	0.818	1.1 (0.5–2.4)	0.747
3	18/207	1.1 (0.5-)2.4	0.827	1.1 (0.5–2.5)	0.765
**Lateral full-thickness loss grade**					
0	17/255	Reference		Reference	
1	25/207	1.8 (0.9–3.4)	0.070	1.9 (1.0-3.6)	0.057
2	12/227	0.8 (0.4–1.7)	0.550	0.8 (0.4–1.8)	0.605
3	7/128	0.8 (0.3-2.0)	0.668	0.9 (0.3-2.0)	0.646
**Lateral lesion sum score**					
From grade 0 to 6	/	0.997 (0.857–1.159)	0.964	0.998 (0.857–1.162)	0.990
**Medial lesion size grade**					
0	3/24	Reference		Reference	
1	5/120	0.3 (0.1–1.5)	0.333	0.3 (0.1–1.5)	0.163
2	28/314	0.7 (0.2–2.5)	0.600	0.8 (0.2–2.8)	0.704
3	25/359	0.6 (0.2-2.0)	0.365	0.6 (0.2–2.1)	0.424
**Medial full-thickness loss grade**					
0	10/139	Reference		Reference	
1	17/282	0.9 (0.4–2.1)	0.867	0.9 (0.4–2.2)	0.903
2	28/275	1.6 (0.7–3.5)	0.217	1.7 (0.8–3.6)	0.193
3	6/121	0.8 (0.3–2.2)	0.622	0.8 (0.3–2.3)	0.676
**Medial lesion sum score**					
From grade 0 to 6	/	1.072 (0.880–1.306)	0.490	1.077 (0.883–1.313)	0.424

OR, odds ratio; CI, confidence interval

※Adjusted for baseline age, sex, BMI and KL grade

### Prediction for 5-year incident surgery

Table 4 presents the PPVs and NPVs of JSN and cartilage lesion features. In general, the PPVs were poor for all the features (similar to the pre-test prevalence of 6.9%). NPVs were high for all the features, among which medial JSN grade had the highest NPV for 5-year incident surgery.

**Table 4:** Predictive values for joint space narrowing and cartilage lesion grades with 5-year incident knee surgery

	Surgery, n	No surgery, n	Positive predictive value (95%CI)	Negative predictive value (95%CI)
**Lateral joint space narrowing grade**				
0	6	103	7% (5 − 9%)	93% (90 − 95%)
Grade 1 or more	55	714		
**Medial joint space narrowing grade**				
0	2	70	7% (6 − 9%)	97% (89 − 99%)
Grade 1 or more	59	747		
**Lateral lesion size grade**				
0	11	138	6% (5 − 9%)	93% (87 − 96%)
Grade 1 or more	50	679		
**Lateral full-thickness loss grade**				
0	17	255	7% (5 − 10%)	94% (90 − 96%)
Grade 1 or more	44	562		
**Medial lesion size grade**				
0	3	24	7% (5 − 9%)	89% (70 − 97%)
Grade 1 or more	58	793		
**Medial full-thickness loss grade**				
0	10	139	7% (5 − 9%)	93% (88 − 97%)
Grade 1 or more	51	678		

CI, confidence interval

## Discussion

In this study, we found no statistically significant association between the presence of JSN or cartilage lesion features observed on patients’ first MRI films and the risk of undergoing knee surgery within a 5-year period. The estimates obtained were small and unlikely to have clinical relevance. Furthermore, these features did not provide any additional predictive value for the occurrence of joint surgery within 5 years.

There are several possible explanations for our findings. Patients with knee OA typically opt for surgical treatment only when the joint becomes severely uncomfortable and significantly impacts their daily lives [14–17]. Among the various symptoms, joint pain is the most common, but previous studies have shown that the presence of cartilage lesions may not be associated with pain, as cartilage does not contain nerves [18]. Studies by Bacon et al. have reported that cartilage thickness loss is only minimally associated with worsening knee pain [19], and this association is partially mediated by synovitis. Therefore, in this study, the cartilage lesions observed on patients’ first MRI films may not cause more severe symptoms, which contradicts the pathway where cartilage lesions lead to increased symptoms and subsequent joint surgery. Additionally, early cartilage lesions do not necessarily indicate future progression of OA. A systematic review by Houck et al. showed that untreated focal chondral defects of the knee joint were not associated with the development of knee OA based on the KL grade within a 2-year follow-up period [20]. Another study by Wluka et al. demonstrated that tibial cartilage loss was not associated with defects in the medial and lateral compartments after 2 years [11]. Furthermore, cartilage loss appears to accelerate only 1–2 years prior to surgery [21]. By assessing the 5-year risk, we may not identify knees that experience cartilage deterioration within a shorter timeframe before surgery. This explains why a similar number of patients in the control group (with no JSN or cartilage lesions) underwent surgery after 5 years.

Our findings diverge from some previous evidence. Cicuttini et al. observed that early tibial cartilage loss over a two-year period independently predicted knee replacement surgery at four years in a study of 113 symptomatic radiographic OA knees [10]. However, the sample size for requiring replacement surgeries was small (*n* = 18), and there were 9 knees with KL grade 1 included in the control group. In the same population, Wluka et al. found that higher baseline total cartilage defect scores were associated with a 6.0-fold increased risk of joint replacement over 4 years [11]. The differences in the study design could explain the inconsistent results, Cicuttini et al. focused on the quantitative changes in cartilage volume within two years, while we focused on baseline cartilage status via a semi-quantitative scoring method. Besides, Sharma et al. studied 841 participants with normal x-rays from the Osteoarthritis Initiative (OAI) cohort and found that cartilage damage at baseline helped predict future incident OA [22]. However, it is important to note that all these studies recruited participants through local advertising from the general population, and the clinical relevance of the baseline timepoint remains unclear, making direct comparisons with our study difficult. Furthermore, the sample sizes for surgery were small in these studies, and they did not consider arthroscopic procedures and tibial osteotomy surgery.

The lack of association between cartilage damage and 5-year surgery rates suggests that slowing cartilage loss may not necessarily reduce the need for surgery. This viewpoint is supported by Eckstein et al. [21] and partially supported by evidence from the FORWARD trial, where increasing cartilage thickness did not result in reduced symptoms within the 2-year timeframe [3]. On the other hand, our findings suggest that there is no need to be overly concerned about cartilage lesions observed on the first MRI films. Clinical physicians should inform patients that these lesions do not necessarily indicate a severe deterioration within the next 5 years that would require surgery. Furthermore, if the patient’s first MRI examination shows no cartilage damage and no significant JSN, it implies a very low probability (around 3%) of undergoing surgery within the next 5 years.

However, this study has some limitations. Firstly, being a retrospective cohort study, selection bias should be admitted since only the patients with MRI data available were included. Besides, we were unable to collect direct evidence on patient symptom information during the follow-up period, which limits our understanding of whether baseline cartilage lesions would result in more severe symptoms in the future. Secondly, there may be potential indication bias since patients who did not undergo MRI examinations were excluded from the study. It is possible that patients who received MRI examinations were more concerned about their health, and clinicians may have paid more attention to those knees. However, it is important to note that most clinically diagnosed OA knees in our hospital typically undergo at least one MRI examination, and the number of exclusions for this reason was small (*n* = 89) in this study. Thirdly, despite including a large group of patients, the overall surgical rate over the 5-year period was low, which may have influenced the predictive values. Fourthly, we determined the JSN and cartilage lesions by using semi-quantitative methods, may have missed minor damages which could be detected by quantitative measurements. Lastly, the results reported in this study specifically pertain to JSN and cartilage lesions identified in the first MRI films of patients and may not be applicable to cases where these features are detected in subsequent examinations.

In conclusion, among symptomatic clinically diagnosed OA knees, cartilage lesions observed in the first MRI examinations were not found to be associated with the occurrence of joint surgery within a 5-year period. The same lack of association was observed for JSN scored on radiographs. All these cartilage-related features appear to have no additional value in predicting 5-year incident joint surgery. However, the absence of these abnormalities, particularly no JSN, may suggest a low risk of undergoing joint surgery within the next 5 years.

### Electronic supplementary material

Below is the link to the electronic supplementary material.

Supplementary Material 1

Supplementary Material 2

## Data Availability

The data underlying this article will be shared on reasonable request to the corresponding author.
