# Supplementary material for: Early cartilage lesion and 5-year incident joint surgery in knee osteoarthritis patients: a retrospective cohort study
Source: BMC Musculoskelet Disord. 2024 May 21;25:398. doi: 10.1186/s12891-024-07225-3 (PMC11106971; doi:10.1186/s12891-024-07225-3)
Supplement: Supplementary file 1 — Supplementary Material 1 [file 12891_2024_7225_MOESM1_ESM.docx]

**Appendix table 1.** Association of baseline Kellgren & Lawrence and joint space narrowing grade with 5-year incident knee surgery after excluding the knees underwent cartilage repair surgery.

|  | Surgery/without surgery | Crude OR  (95% CI) | P values | Adjusted OR  (95% CI) | P values |
| --- | --- | --- | --- | --- | --- |
| Lateral joint space narrowing grade |  |  |  |  |  |
| 0 | 5/103 | Reference |  | Reference |  |
| 1 | 39/506 | 1.6 (0.6-4.1) | 0.343 | 1.6 (0.6-4.1) | 0.342 |
| 2 & 3 | 11/208 | 1.1 (0.4-3.2) | 0.877 | 1.1 (0.4-3.2) | 0.891 |
| Medial joint space narrowing grade |  |  |  |  |  |
| 0 | 1/70 | Reference |  | Reference |  |
| 1 | 43/525 | 5.7 (0.8-42.3) | 0.087 | 5.8 (0.8-42.6) | 0.085 |
| 2 & 3 | 11/222 | 3.5 (0.4-27.3) | 0.238 | 3.4 (0.4-27.1) | 0.241 |

OR, odds ratio; CI, confidence interval.
