# Supplementary material for: Early cartilage lesion and 5-year incident joint surgery in knee osteoarthritis patients: a retrospective cohort study
Source: BMC Musculoskelet Disord. 2024 May 21;25:398. doi: 10.1186/s12891-024-07225-3 (PMC11106971; doi:10.1186/s12891-024-07225-3)
Supplement: Supplementary file 2 — Supplementary Material 2 [file 12891_2024_7225_MOESM2_ESM.docx]

**Appendix table 2.** Association of baseline tibiofemoral cartilage lesion grade with 5-year incident knee surgery after excluding the knees underwent cartilage repair surgery.

|  | Surgery/without surgery | Crude OR/β  (95% CI) | P values | Adjusted OR/β  (95% CI)※ | P values |
| --- | --- | --- | --- | --- | --- |
| Lateral lesion size grade |  |  |  |  |  |
| 0 | 10/138 | Reference |  | Reference |  |
| 1 | 10/231 | 0.6 (0.2-1.5) | 0.263 | 0.6 (0.3-1.5) | 0.295 |
| 2 | 19/241 | 1.1 (0.5-2.4) | 0.835 | 1.1 (0.5-2.5) | 0.790 |
| 3 | 16/207 | 1.1 (0.5-2.3) | 0.877 | 1.1 (0.5-2.5) | 0.822 |
| Lateral full-thickness loss grade |  |  |  |  |  |
| 0 | 16/255 | Reference |  | Reference |  |
| 1 | 22/207 | 1.7 (0.9-3.3) | 0.123 | 1.7 (0.9-3.4) | 0.106 |
| 2 | 11/227 | 0.8 (0.4-1.7) | 0.521 | 0.8 (0.4-1.7) | 0.556 |
| 3 | 6/128 | 0.7 (0.3-2.0) | 0.552 | 0.7 (0.3-1.9) | 0.539 |
| Lateral lesion sum score |  |  |  |  |  |
| From grade 0 to 6 | / | -0.013 (-0.172-0.146)# | 0.987 | -0.011 (-0.168-0.146)# | 0.892 |
| Medial lesion size grade |  |  |  |  |  |
| 0 | 2/24 | Reference |  | Reference |  |
| 1 | 4/120 | 0.4 (0.1-2.3) | 0.306 | 0.4 (0.1-2.4) | 0.323 |
| 2 | 26/314 | 1.0 (0.2-4.4) | 0.993 | 1.1 (0.2-4.8) | 0.927 |
| 3 | 23/359 | 0.8 (0.2-3.5) | 0.732 | 0.8 (0.2-3.7) | 0.790 |
| Medial full-thickness loss grade |  |  |  |  |  |
| 0 | 8/139 | Reference |  | Reference |  |
| 1 | 15/282 | 0.9 (0.4-2.2) | 0.861 | 0.9 (0.4-2.3) | 0.884 |
| 2 | 26/275 | 1.6 (0.7-3.7) | 0.234 | 1.7 (0.7-3.8) | 0.216 |
| 3 | 6/121 | 0.9 (0.3-2.5) | 0.788 | 0.9 (0.3-2.6) | 0.834 |
| Medial lesion sum score |  |  |  |  |  |
| From grade 0 to 6 | / | 0.089 (-0.119-0.297)# | 0.399 | 0.098 (-0.110-0.306)# | 0.355 |

OR, odds ratio; CI, confidence interval.

※Adjusted for baseline age, sex and BMI; #presents β (95%CI).
